# Autism Spectrum Disorder: The Impact of Stressful and Traumatic Life Events and Implications for Clinical Practice

**DOI:** 10.1007/s10615-018-0649-6

**Published:** 2018-01-17

**Authors:** Samantha Fuld

**Affiliations:** NYU Silver School of Social Work, New York University, 1201 West Mount Royal Avenue #304, Baltimore, MD 21217 USA

**Keywords:** Autism spectrum disorder, Trauma, Comorbidity, Adverse childhood experiences, Mental health, Developmental disabilities

## Abstract

Research findings suggest that behavioral interventions are effective in improving educational outcomes and fostering skill development in people with autism spectrum disorder (ASD). However, high rates of comorbidity between ASD and other psychological disorders, including depression and anxiety, indicate that standard behavioral approaches are not adequately addressing issues related to mental health in this population. Research emerging since the publication of the fifth edition of the Diagnostic and Statistical Manual of Mental Disorders (DSM-5) is advancing our understanding of the nature of childhood stress and trauma in people with ASD and its subsequent impact on mental health and wellbeing. Mounting evidence for stress and trauma as a risk factor for comorbidity and the worsening of core ASD symptoms may intimate a shift in the way clinical social workers and other clinical practitioners conceptualize and approach work with this population to include trauma-focused assessment strategies and clinical interventions. Future directions for research to better understand the nature of childhood stress and trauma and improve mental health in this population are also discussed.

## Introduction

Particularly in North America, professionals, including social workers, are taught to support people with autism spectrum disorder (ASD) and their families through primarily behavioral interventions (Keenan et al. 2015). Research indicates that such interventions are effective in improving educational outcomes and fostering various types of skill development (Fonagy et al. 2015; Peters-Scheffer et al. 2011). However, high rates of comorbidity between ASD and other psychological disorders indicate that standard behavior therapies are not adequately addressing issues related to mental health and wellbeing. Since the publication of the fifth edition of the Diagnostic and Statistical Manual of Mental Disorders (DSM-5) (American Psychiatric Association 2013), the symptomatic features and risk factors associated with comorbidity in individuals with ASD have emerged as important new areas of research. Preliminary findings indicate that people with ASD may be at high risk for experiencing stressful and traumatic life events, the sequelae of which can negatively impact mental health through the development of comorbid psychopathology and/or worsening of the core symptoms of ASD (Mehtar and Mukaddes 2011; Taylor and Gotham 2016). These findings are consistent with previous research on the psychological consequences of adverse childhood experiences (ACEs) in the general population (Anda et al. 2006; Edwards et al. 2003; Felitti et al. 1998). Importantly, it is possible that the nature of ASD may enhance vulnerability to trauma and its sequelae (Kerns et al. 2015).

The purpose of this paper is to review emerging research on traumatic and stressful life events as they impact mental health in individuals with ASD. The significance of this research suggests the need for a shift in the way social work practitioners conceptualize and approach work with this population, such that the presence and impact of stress and trauma is considered as part of the assessment and treatment planning process. Future directions for research to (1) better understand the risk and resilience factors associated the impact of stress and trauma on mental health in people with ASD and (2) develop effective assessment strategies and trauma-focused clinical interventions to improve mental health in this population will also be discussed.

## Review of the Literature

### Autism Spectrum Disorder

Autism spectrum disorder as a diagnosis is new to the DSM-5 (APA 2013). It represents an effort to combine what were previously identified as several distinct but related disorders impacting social communication and development into one diagnostic spectrum with three levels that denote severity based on needed support. ASD is classified as a neurodevelopmental disorder (APA 2013). It is characterized by difficulty with social communication as evidenced by “deficits in social-emotional reciprocity… deficits in nonverbal communicative behaviors for social interaction…[and] deficits in developing, maintaining and understanding relationships” (APA 2013, p. 50). Additionally, people with ASD have some form of restricted and/or repetitive behaviors which can include: repetitive speech or movements; strong desire for sameness, predictability and routine; intense or unusually specific interests; and hypo or hyper sensitivity to sensory experiences such as lights, sounds and physical sensations. These symptoms present in different combinations and with great variability in their presentation and impact on a person’s daily functioning (APA 2013).

In plain language, people with ASD are sometimes described as literal, direct, honest, persistent and loyal (Atwood 2008; Grandin 2011). They may be uncomfortable with eye contact and prefer certain textures of food or clothing. When overwhelmed by strong emotions, including anxiety and boredom, some people with ASD may exhibit repetitive “stimming” behavior such pacing, flapping or verbally repeating a certain word or phrase (Sinha et al. 2014). Many people with ASD are distressed by unanticipated schedule changes or unexpected events and tend to thrive with rules and routine (Grandin 2011). The detailed and logical thinking sometimes associated with ASD can be a significant strength in some employment contexts including but not limited to computer programming, library sciences and data analytics (Lee and Carter 2012). While overall these descriptors may represent some common features, ASD is known to be an exceptionally heterogenous diagnosis. One study captured this, describing, “you could get fifty kids with the same [ASD] diagnosis and they are all completely different” (Cage et al. 2016, p. 18). For clinicians unfamiliar with ASD, this variability can make identification of ASD symptoms and differential diagnosis challenging (Mogro-Wilson et al. 2014).

Autism spectrum disorder severity is determined based on the level of support a person needs in the realms of social communication and restrictive or repetitive behaviors (APA 2013). In research however, the presence or absence of intellectual disability (ID) and the extent of cognitive impairment is sometimes used to estimate ASD severity. ID and ASD co-occur in some individuals, though Charman et al. (2011) point out that the rates of ID in people with ASD are lower than previously thought. A study conducted by the Centers for Disease Control and Prevention’s (CDC) Autism and Developmental Disabilities Monitoring Network (Baio and CDC 2012) reported that 38% of the children with ASD at their monitoring sites in 2008 also had an ID. Another epidemiological study conducted by Charman et al. (2011) reported that just over half of their sample of children with ASD also met criteria for ID. Of those who did meet criteria for ID, most fell into the category of “mild” severity. Importantly Robertson (2009) points out that ID is sometimes wrongly conflated with a person with ASD’s verbal abilities, noting that the cognitive capabilities of people with ASD who are nonverbal or minimally verbal are often underestimated, particularly as many do not have adequate access to assistive technology.

Estimates of ASD prevalence have risen steadily over the past 2 decades. According to a CDC report in 2014 (CDC 2014), which analyzed diagnostic data on 8-year-old children from 2010, current estimates of ASD prevalence are 1 in 68, compared to 1 in 150 in 2000. The underlying cause of ASD and factors contributing to its increased prevalence are not well understood (APA 2013; Fakhoury 2015). Questions remain as to the amount of rise that can be attributed to increasingly sophisticated diagnostic screening tools identifying cases of ASD which would previously have been undetected. Currently it is believed that genetic, epigenetic and purely environmental factors all may contribute to the etiology ASD as well as to its increased prevalence (Fakhoury 2015; Hertz-Picciotto et al. 2006). Given the high incidence of ASD, it is likely that social workers in a number of settings such as schools, clinics, hospitals, shelters and the child welfare system will encounter people with ASD and their families.

### Mental Health and Comorbidity in ASD

The mental health of people with ASD is an important area of concern, particularly for clinical social workers. Comorbidity in ASD refers to those who meet diagnostic criteria for one or more mental health disorders in addition to their ASD diagnosis (Mannion et al. 2014). Comorbidity research in ASD is in its nascent stages, as comorbidities between ASD and other mental health disorders have only recently been acknowledged within the psychological community (Kerns et al. 2016; Matson and Williams 2014). What is known, is that comorbidity in people with ASD seems to be more the norm than the exception (Reinvall 2016). According to the DSM-5, approximately 70% of individuals with ASD have one comorbid mental health disorder and up to 40% may have two or more (APA 2013). Mannion and Leader (2013) conducted a literature review on comorbidity in children and adults with ASD and concluded that overall evidence suggests significant levels of comorbidity with depressive disorders, anxiety disorders, and ADHD. Suicidal ideation (SI) has also been shown to be higher amongst children and adults with ASD than in the general population (Matson and Williams 2014). A study by Mayes et al. (2013) found that in a sample of more than 700 children with ASD, SI and/or an attempt were 28 times higher than for neurotypical peers. Though rates of SI among neurotypical youth with underlying psychopathology including depression and anxiety may be comparable to or higher than those with ASD (Mayes et al. 2013; Storch et al. 2013). Unfortunately, it is difficult to draw firm conclusions as SI often accompanies a variety of comorbidities including depression, anxiety, behavioral problems and posttraumatic stress disorder (PTSD) in people with ASD and is an area that is not well researched or understood (Storch et al. 2013). Clearly, future research to better understand the rates of and characteristics of comorbidity is required, as significant variability is reported across studies (Mannion and Leader 2013; Reinvall 2016).

The research findings regarding any relationship between ASD severity and comorbidity remain ambiguous. Reinvall et al. (2016) reported that some studies have identified higher rates of comorbid depression and anxiety in those with “higher functioning” ASD, noting that this finding is often attributed to the fact that individuals who are higher functioning are more aware of their social differences than those with more severe ASD symptoms. However, the authors note that studies conducted via interview as opposed to questionnaire tend to show no difference in comorbidity among those with varying IQ or varying levels of ASD severity. Mannion et al. (2014) also note that people with more severe ASD, particularly those who have difficulty communicating, tend to receive diagnoses of comorbid psychopathology less often as differential diagnosis can be harder to assess. Even without communication barriers they note that differential diagnosis of comorbidity in ASD can be challenging as core symptoms of ASD such as rigidity, rumination and social withdrawal can also be symptoms of various mental health disorders including anxiety and depression (Mannion et al. 2014).

While additional research is required on multiple levels to fully understand the complexities of comorbidity in ASD, one area that has been notably overlooked is posttraumatic stress disorder (PTSD) and other trauma and stressor-related disorders (Reinvall et al. 2016). A recent study by Roberts et al. (2015) found a strong association between trauma, PTSD and *autistic traits* (which may have been sub-clinical) in adult women. This association was highest amongst those women with the most severe autistic traits. However, as this study was conducted using retrospective data, clinical diagnosis could not be confirmed. Mehtar and Mukaddes (2011) examined the presentation of PTSD in children and adolescents with ASD and found that some of the PTSD symptoms that presented in their sample (e.g. aggressive outbursts, distractibility, social isolation) could be mistaken as an exacerbation of ASD symptoms. As a result, these authors recommended that comprehensive assessment, including a detailed trauma history be conducted when people with ASD present for clinical treatment (Mehtar and Mukaddes 2011).

### ASD and the Impact of Traumatic and Stressful Life Events

Due to differences among researchers in the way the constructs of stressful and traumatic life events are defined and sometimes conflated, it is necessary to consider both when examining the impact of such experiences on mental health in people with ASD. The DSM-5 distinguishes between “traumatic and stressful event(s)” (APA 2013, p. 265), defining trauma as “exposure to actual or threatened death, serious injury or sexual violence” (p. 271). Stressful events and stressors represent a broader category and can be associated with loss, work, relationships, one’s environment, life transitions, medical or physical struggles, and perceived lack of achievement (APA 2013). The DSM-5 notes that psychological distress associated with stress and trauma is varied and may include anxiety or fear-based reactions, changes in mood, anger, irritability, aggression or dissociation. Although there is a specific diagnostic category for trauma and stressor-related disorders, stress and trauma are identified as risk factors for several other disorders including depression and anxiety (APA 2013).

An important development in understanding the impact of stress and trauma on mental health in the general population has been the adverse childhood experience (ACE) studies. This growing body of literature suggests a strong relationship between traumatic or stressful events in childhood (ages 0–18) and psychopathology (Schilling et al. 2007). Berg et al. (2016) recently undertook the first study to identify rates of ACEs in children with ASD. Results from this study suggest that a diagnosis of ASD is significantly associated with a higher probability of reporting one or more ACEs. Additionally, the number of children with ASD who were exposed to four or more ACEs was twice as high as neurotypical peers. Given these findings, Berg et al. (2016) suggest that children and adults who have been exposed to stressful and potentially traumatic life events are at risk for the development of subsequent mental health disorders.

While studies have documented the impact of certain stressful events on children with ASD, there is reason to believe that there may be a stronger relationship when the field of potentially adverse experiences is expanded. The questionnaire used to assess ACEs in the study by Berg et al. (2016), which was conducted using data from the National Survey of Children’s Health, focused on situational indicators of stress and trauma experienced by the family. These included family instability, traumatic loss, poverty and neighborhood violence. The questionnaire utilized in this study did not pose direct questions about experiences of verbal, physical or sexual abuse unrelated to neighborhood violence. Additionally, as the data collected was not specific to this study, it did not include questions regarding ACEs that might be uniquely relevant for this population. For example, the questionnaire did not inquire about adverse social experiences such as peer-victimization, which are known to be significantly higher among youth with ASD than typically developing peers, and like other ACEs are also known to be a risk factor for the development of mental health problems (Cappadocia et al. 2012; Zeedyk et al. 2014). Although participants were asked if the child was “treated or judged unfairly due to race/ethnicity” (Berg et al. 2016, p. 1125), they were not asked if the child was ever treated or judged unfairly due to their ASD diagnosis or presentation of ASD symptoms. Future research that includes the examination of ACEs specific to ASD would be important to gain a clearer understanding of the full range of stressors facing children with ASD.

Kearns et al. (2015) further illuminate common experiences specific to children with ASD that may be important to consider when evaluating stress and trauma. Offering a somewhat different definition of trauma than the one outlined in the DSM-5, Kearns et al. (2015) focus on an individual’s perception of the event, describing trauma more broadly as “an event that damages or harms the individual even though the severity, longevity and permanency of that harm may vary widely” (p. 3475). The authors discuss the possibility that the core symptoms of ASD may predispose children to stressful experiences. For example, difficulty with socialization could lead to increased social anxiety (Bellini 2006) or peer rejection. They also suggest that experiences known to be distressing for people with ASD such as unexpected schedule changes, the prevention or discouragement of repetitive or preferred behaviors, and certain sensory sensitivities, could be perceived as traumatic particularly when such distress occurs on a regular basis, adding to the potential for comorbidity (Kerns et al. 2015). Such a conclusion is theoretical and requires further research. However, there is some biological evidence supporting the theory that people with ASD do experience exaggerated and/or altered stress responses.

One of the primary systems involved in the physiological response to stress in humans is the hypothalamic–pituitary–adrenal (HPA) axis (Taylor and Corbett 2014). This system regulates the release of cortisol, a hormone associated with both the physiological and psychological experience of stress. There are two different ways that researchers have sought to understand the physiological stress response in people with ASD. One area of research has examined whether common changes exist in the regular daily rhythms of cortisol secretion (Sivaratnam et al. 2015). In most people, daily cortisol levels follow a general pattern, increasing significantly in the morning and then decreasing throughout the day. There is some evidence to suggest that this pattern may be disrupted in some people with ASD, particularly those with more severe ASD symptoms (Taylor and Corbett 2014). However, a specific altered pattern has not been found, possibly due to the significant heterogeneity within the autism spectrum (Taylor and Corbett 2014). Despite the absence of a particular alteration in daily cortisol rhythms, some type of dysregulation of the HPA axis in people with ASD has been a common finding across the literature (Sivaratnam et al. 2015).

While findings on changes in daily cortisol rhythms have been ambiguous other than to suggest some kind of general dysregulation, other research has examined physiological stress in people with ASD in response to specific stimuli. Taylor and Corbett (2014) reviewed literature on specific stressors, finding evidence of hyperarousal of the HPA axis in response to common social interactions, novel situations, and unpleasant or painful stimuli. These authors concluded, “many persons with ASD exhibit marked stress responses in otherwise benign, novel and social situations. The hyper-responsivity may contribute to increased anxiety, neophobia, or even chronic stress” (p. 225). Spratt et al. (2012) also note a delayed response of the HPA axis in halting cortisol secretion following what is perceived as a negative stressor, indicating that children with ASD may not only experience more stress than neurotypical children in response to certain situations, but their body’s stress response may be longer lasting. This is echoed by Sivaratnam et al. (2015) who point out that when looking at the overall body of research on cortisol responsiveness in children with ASD, findings indicate, “a generalised sensitivity to both emotional and non-emotional stressors” (p. 231). While most of this research has focused on children, it is also important to consider that the common stressors experienced by children with ASD are unlikely to abate in adulthood. Bishop-Fitzpatricket al. (2015) found that adults with ASD experience significantly greater overall stress than neurotypical adults. These findings are relevant as ongoing exposure to elevated cortisol is known to negatively impact both physical and mental health (Taylor and Corbett 2014). Specifically, Mahar et al. (2014) point out that chronic dysregulation of the HPA axis in response to stress (as is seen in people with ASD) can have neurotoxic effects, potentially predisposing people to a number of psychological and behavioral stress reactions including depression (Mahar et al. 2014), anxiety, and PTSD (Danese and McEwen 2012; Sharpley et al. 2016).

Further considering the psychological impact of stress and trauma on people with ASD, Taylor and Gotham (2016) conducted a study of young adults with ASD (ages 17–22) that examined “major and potentially traumatic life events” (p. 4), and clinical symptoms of mood and anxiety disorders. The life events included in the survey encompassed those that would be considered traumatic in the DSM-5 (i.e. various types of abuse, serious injury, etc.) and those that would be identified as stressful (i.e. challenges in relationships, being held back in school, etc.). Importantly, the parents of young adults with ASD who participated in this survey reported not only whether each event occurred, but the extent to which their child seemed affected by the event. Parent report of the impact of the event on their child served as a proxy measure for whether the event was perceived by the child as “traumatic.” Notably, this study was designed to capture very conservative estimates of perceived trauma. Respondents needed to indicate an *extreme* level of distress in response to the event for it to be considered traumatic. The results indicated no significant association between trauma and clinical symptoms of anxiety. However, approximately 90% of individuals with clinically relevant mood symptoms were reported to have experienced one or more traumatic events in childhood. Among those without comorbid mood symptomatology, only 40% reported trauma. The researchers reported that this finding was consistent when controlling for IQ and summarized that “although a significant portion of youth with trauma do not have a co-occurring mood disorder, these disorders were rarely observed in the absence of an event that is experienced as traumatic” (p. 7).

## Discussion

The studies by Berg et al. (2016) on the prevalence of ACEs in children with ASD and by Taylor and Gotham (2016) on trauma and psychopathology in young adults with ASD present compelling new research indicating that children with ASD may be more likely to experience traumatic and stressful life events than their typically developing peers. Evidence supports this not only qualitatively, but also on a physiological level given the findings of hyperarousal and general dysregulation of the HPA axis in response to negative stressors, social interactions and new or unexpected experiences (Spratt et al. 2012; Taylor and Corbett 2014). Stressful and traumatic events are known to increase the risk for negative mental health outcomes in the general population (Anda et al. 2006; Edwards et al. 2003; Schilling et al. 2007) and the research reviewed in this article offers some convincing indications that this is likely to be the case for those with ASD as well (Taylor and Gotham 2016).

Importantly, there are both strengths and limitations to the current research. The studies by Berg et al. (2016), Taylor and Gotham (2016) and in fact much of the existing research related to both trauma and mental health in ASD relies heavily on caregiver report. The benefit of caregiver report as Taylor and Gotham (2016) point out, is that it allows the inclusion of people with greater communication difficulties who may not be able to independently complete self-report measures or traditional clinical interviews. However, these individuals may also have difficulty communicating internal subjective experiences to their caregivers (Mehtar and Mukaddes 2011). Subjective experience is important in assessing whether an event was experienced as traumatic. Thus, without obtaining this information directly from the individuals with ASD, conclusions about their internal experience of the event only represents informed conjecture.

There are also questions regarding the generalizability and strength of these findings. While Taylor and Gotham’s study (2016) had a relatively small sample, each participant received gold-standard diagnostic screening to determine their ASD diagnosis, IQ and clinical mood/anxiety symptoms. Berg et al. (2016) conducted a large-scale study utilizing data from the National Survey of Children’s Health, which provided a robust sample, but diagnosis of ASD and ASD severity were based on parent report and could not be confirmed. Despite these limitations, both studies provide important insights and seem to confirm prior studies related to the experience of stress and trauma, such as those discussed by Kerns et al. (2015). Additionally, Taylor and Gotham’s (2016) findings demonstrating a relationship between mood symptomatology and traumatic life events in people with ASD are consistent with what is known about the impact of ACEs in neurotypical populations (Anda et al. 2006; Edwards et al. 2003; Felitti et al. 1998).

One question that needs to be further addressed in the research is the issue of diversity within the autism spectrum and how individual characteristics, presentation, and severity may mediate exposure to stress and trauma and their sequelae. Importantly, both Berg et al. (2016) and Taylor and Gotham (2016) included participants with ASD across the spectrum of functioning in their samples. Berg et al. (2016) found that greater severity in ASD symptoms was associated with higher ACE scores, though those with “mild” ASD also had significantly higher ACE scores than their typically developing peers. Conversely, Taylor and Gotham (2016) found no significant differences in rates of traumatic experiences or clinical symptomatology when adjusting for severity, albeit this was measured by IQ which may represent a slightly different construct. One possible reason for this discrepancy is that the survey utilized by Taylor and Gotham (2016) included more of what the DSM would consider *stressful life events*, such as relational problems, bullying and failure in school. Such events were not included to the same extent in the ACE questionnaire utilized by Berg et al. (2016), which was briefer and inquired about events representing a closer fit to the narrower DSM-5 definition of trauma (though not all would fit that definition). Such an explanation could indicate differences in the types of stressful and traumatic life events experienced by people with differing levels of ASD severity. Exposure to different types of stressful and traumatic events may also lead to different common sequelae and thus is an important area for further inquiry.

As may be evident from the prior discussion of various terms and definitions associated with traumatic and/or stressful life events and the ways that different researchers attempt to capture these constructs, there is debate about what constitutes stress and trauma, particularly it relates to people with ASD. Mehtar and Mukaddes (2011) point out that people with ASD may have difficulty describing internal experiences, feelings and reactions and thus it can be a challenge to determine levels of distress associated with traumatic or stressful events. Furthermore, one could hypothesize that the nature of ASD, specifically the lack of attention to external social experiences, may serve as a protective factor in relation to adverse social experiences such as peer-victimization or the recognition of stigma associated with an ASD diagnosis. Given the diversity and variation within the autism spectrum, such a hypothesis could have relevance for some. However, there is research suggesting that the social difficulties associated with ASD can be quite distressing for some individuals. Studies have reported that adolescents with ASD experience and can be distressed by feelings of loneliness (Locke et al. 2010), ostracism (Sebastian et al. 2009) and negative perceptions of what it means to have ASD (Humphrey and Lewis 2008). High levels of both gelotophobia (fear of being laughed at) (Wu et al. 2015) and social anxiety (Bellini 2006) have also been reported in adolescents with ASD. Much of this research has been conducted on adolescents with ASD who are relatively high-functioning so it may not be generalizable across the spectrum. However, these findings do indicate that some people with ASD are not only aware of their social difficulties, but also care deeply and worry about it. Though adapting their social behavior to match that of their peers can be challenging for children with ASD (Kerns et al. 2015), and may thus be a source of stress.

Another interesting area to further explore is the suggestion by Kerns et al. (2015) that the core symptoms of ASD may themselves predispose children to stressful and traumatic experiences. They discuss this in terms of the social challenges and difficulty adjusting to unexpected circumstances associated with ASD. Logically, these core symptoms would make it likely that navigating everyday social situations and new or unexpected experiences could be perceived as stressful by someone with ASD. As Kerns et al. (2015) indicate, the repeated nature of these demands from the environment could indeed be interpreted or experienced by some individuals with ASD as chronically stressful or potentially even traumatic over time. This suggestion is supported by the research finding hyperresponsiveness of the HPA axis in response to exactly these types of demands (common social interactions, novel situations, and unpleasant stimuli) (Taylor and Corbett 2014). Interestingly, Storch et al. (2013) point out that a similar process could be involved in the development of some types of comorbid mental health symptoms, including depression, PTSD, and SI.

Both Storch et al. (2013) and Mayes et al. (2013) examined the risk factors associated with SI in children with ASD. Storch et al. (2013) identified depression and PTSD as some of the most significant risk factors for SI in this population. Mayes et al. (2013) also identified depression as a primary risk factor, as well as several additional social risk factors including: peer-victimization, race (being Black or Hispanic) and socioeconomic disadvantages (which are known risk factors for SI in typically developing children as well). Although the process underlying SI in children with ASD remains unclear (Hannon and Taylor 2013), Storch et al. (2013) propose that the repetitive and ruminative nature of the thought process in some people with ASD may serve to enhance SI. Using this theoretical model, a child with ASD experiencing challenging or stressful life events may be predisposed to ruminate on negative experiences, including the psychosocial stressors such as peer-victimization identified by Mayes et al. (2013). It is possible that consistent rumination on stressful or traumatic experiences could lead to symptoms of depression, anxiety or even PTSD if a significant traumatic event has taken place. Storch et al. (2013) additionally point out that people with ASD often experience rigidity in their thinking and may assume that challenging thoughts, feeling and situations will be permanent. Cognitive theory would suggest that negative thought patterns including depression and SI could develop from such beliefs that negative experiences are likely to be permanent (Beck 2011).

An additional explanation might be considered when examining differences in rates of SI within the ASD population. Mayes et al. (2013) found no significant differences in rates of SI when accounting for IQ, however Storch et al. (2013) did find that among people with ASD without co-occurring ID, those with greater social and communication impairment exhibited higher rates of SI. This is perhaps unsurprising as these individuals were likely to be aware of their ASD symptoms but less able to compensate for them and *fit in* as someone with milder social impairment may be able to do. Ultimately, this could conceivably lead to greater degrees of psychological distress as evidenced by higher rates of developing SI. Such theoretical explanations of course require additional research. People with ASD who experience SI are an extreme example of how stressful and traumatic experiences may interact with ASD symptoms and have the potential to influence mental health. However, when considered holistically, the current research lends enough support to these hypotheses to make them worthwhile for clinical social workers to consider when working with people with ASD.

The research presented in this paper offers a concerning perspective on the frequency that children with ASD may experience traumatic or stressful life events and the potential for such events to negatively impact mental health. However, this matter is complex and such findings should not be generalized to every person with ASD. While it appears that traumatic and stressful life events may occur more frequently for children with ASD, experiencing these events is by no means a certainty. In the study by Berg et al. (2016) just over half of children with ASD were reported to have experienced at least one ACE, meaning that based on parent reports the other half did not. In contrast, all respondents in the study by Taylor and Gotham (2016) reported exposure to at least one traumatic or stressful life event, however this questionnaire included a broader range of events. Importantly, of those who reported experiencing each event, very few events were described as traumatic by every respondent. The only events that were reported as traumatic by 100% respondents were: living apart from one or both parents, someone in the home dying by suicide, and witnessing any form of abuse (Taylor and Gotham 2016). Furthermore, while a majority of those presenting with mood symptoms had experienced a traumatic event, 40% of those who experienced trauma did not present with clinically significant symptoms of a mood disorder (Taylor and Gotham 2016). This indicates the presence of resilience factors that are an important area of inquiry for future research.

## Implications for Practice

The emerging research on traumatic and stressful life events in people with ASD engenders many questions and requires deeper investigation. However, the implications of this research, particularly regarding mental health and wellbeing, make it important for clinical social workers to be aware of these preliminary findings. Unfortunately, Laws et al. (2010) point out that clinical professionals currently receive very little training to work with an ASD population beyond standard behavioral approaches. This is particularly the case as it relates to mental health (Robertson 2009). Current evidence-based treatment approaches tend to focus overwhelmingly on reducing negative social, behavioral and academic impacts of the core symptoms of ASD (Wong et al. 2015). In fact, in a systematic review of evidence-based practices for youth with ASD, out of 456 intervention articles identified by the authors for inclusion in their study, only one focused on outcomes related to mental health and wellbeing (Wong et al. 2015). Strategies to address the experience of trauma and its subsequent impact on mental health are notably absent. Supporting these findings, Fonagy et al. (2015) identify, “promotion of normal development,” “reduction of rigidity,” “elimination of nonspecific maladaptive behaviors,” and “alleviation of family distress” (p. 276) as the most common treatment goals in clinical work related to ASD. Based on this information, one might argue that issues related to trauma and mental health seem to be overlooked within common treatment frameworks for ASD.

Alternatively, considering the suggestion Kerns et al. (2015) and Storch et al. (2013) that the core symptoms of ASD may predispose a person to stressful and traumatic life experiences as well as their sequalae, one might conclude that focusing clinical interventions on the core symptoms of ASD to prevent and potentially address negative mental health outcomes associated with stress and trauma may seem promising. However, from a pragmatic perspective it could be argued that this is already happening and does not seem to be effective. Most of the current models for treatment and early intervention already focus on the core-symptoms of ASD and do so with some efficacy (Peters-Scheffer et al. 2011; Wong et al. 2015), so it seems unlikely based on the high rates of trauma/stress and comorbidity in ASD that standard treatment approaches are effective in this realm.

Given the relationship between stressful life events and ASD symptoms, it is important for social workers to identify these risk factors when making a diagnosis. Research indicates that stressful and traumatic life events are an underlying risk factor for virtually all the comorbid psychopathologies that are common in people with ASD (APA 2013; Mannion et al. 2014). Additionally, exposure to stressful and potentially traumatic events may manifest as symptoms of aggression, difficulty concentrating, social isolation, increased relational difficulties, regression in daily living skills, and increased repetitive or stereotypic behavior (Bishop-Fitzpatrick et al. 2015; García-Villamisar and Rojahn 2015; Mehtar and Mukaddes 2011). As many of these symptoms are commonly associated with ASD, they may be assessed by professionals as part of the ASD diagnosis, meaning that the stress and/or trauma underlying these symptoms remains untreated (Kearns et al. 2015). Thus, it is critical that clinical social workers keep this in mind when conducting assessments, functional assessments, engaging in case consultations, and contributing to the treatment and treatment planning. While validated clinical tools to assess traumatic and stressful life events in people with ASD do not currently exist, assessment scales such as those utilized in the research studies on ASD and trauma (Berg et al. 2016; Mehtar and Mukaddes 2011; Taylor and Gotham 2016) could be useful tools to aid clinicians in conducting trauma-focused assessments with individuals with ASD.

Given the dearth of research on trauma-informed treatment strategies for people with ASD, clinicians who work with clients with ASD who are exhibiting symptoms associated with or exacerbated by traumatic or stressful life events are left without guidance. While trauma-informed treatment models have not been widely studied in individuals with ASD, Harvey (2012) has written about strategies to address trauma in people with ID, which may have some relevance for those with ASD as well. In addition to addressing trauma associated with peer-victimization and abuse, taking a similar approach to Kerns et al. (2015), Harvey highlights the importance of addressing trauma associated with the experience of a person’s ID (or in this case ASD) diagnosis. Harvey (2012) describes the “trauma of invalidation” (p. 57), that can occur when a child feels invalidated by their social environment. In ASD this could certainly occur through social rejection or abuse, and could also perhaps occur when it is communicated to a child that something like a schedule change or an unpleasant texture that may be highly distressing for that individual, should not be so upsetting. Within treatment, Harvey (2012) suggests working with clients on themes associated with regaining a sense of power, a sense of connection to others, and a sense of both physical and emotional safety. It is possible that some of these themes suggested by Harvey could lend themselves to cognitive-behavioral interventions, which is one of the psychotherapeutic models that has been applied most often in treating mental health concerns in individuals with ASD, though these interventions have typically been focused on anxiety (Fonagy et al. 2015; Mannion et al. 2014).

Similarly, Danese and McEwen (2012) suggest that neurotypical children who develop similar patterns of HPA activation and dysregulation as those with ASD as a result of, “chronic exposure to psychosocial stressors” (p. 30), may see a lessening in dysregulation of the HPA axis when placed in a supportive, nurturing and affirming environment. While for those with ASD, it may not be possible to remove all significant environmental stressors as some are common to the everyday environment, this information could inform treatment goals and intervention strategies that focus not only on maladaptive behaviors and reducing rigidity, but promoting an environment that is supportive and affirmative of the experience of having ASD. Importantly, Cage et al. (2016) note that while some teens with ASD experience concern and distress regarding the way their peers perceive them, they also found that some take pride in their autistic identity and uniqueness from their peers. While further research is needed to confirm this finding, such an attitude may represent an important resilience factor and certainly has implications for clinical treatment and prevention strategies that promote a neurodiversity perspective and help to create an affirmative environment that facilitates healthy coping with common stressors and supports mental health (Cage et al. 2016; Robertson 2009).

## Conclusion

This paper explores emerging research associated with traumatic and stressful life events as they impact mental health and wellbeing in people with ASD. While this area clearly requires further research, the preliminary findings raise important questions for clinical social workers and other mental health professionals as to how we conceptualize the issues facing those with ASD and ultimately how we practice. Stressful and traumatic life events should be considered by clinical practitioners when conducting assessments and determining appropriate treatment plans for people with ASD experiencing comorbid symptomatology and or/an exacerbation of core ASD symptoms to help ensure that underlying causes of these symptoms are not overlooked.
